# Use of Face-to-Face Assessment Methods in E-Learning—An Example of an Objective Structured Clinical Examination (OSCE) Test

**DOI:** 10.3390/pharmacy9030144

**Published:** 2021-08-20

**Authors:** Kristiina Sepp, Daisy Volmer

**Affiliations:** Institute of Pharmacy, Faculty of Medicine, University of Tartu, 50090 Tartu, Estonia; kristiina.sepp@ut.ee

**Keywords:** pharmacy education, OSCE, e-learning, digital health training, Estonia

## Abstract

The spread of COVID-19 and social-distancing rules have increased the need for alternative learning environments with a focus on e-learning platforms. The objective of this study was to assess whether and to what extent the transition from traditional learning and assessment environment to the e-setting impacts the knowledge and skills acquired by students and their satisfaction with new e-solutions of taking the OSCE test. The study compared the results of three face-to-face (2018–2019) and one electronically conducted (2021) OSCE tests, as well as students’ feedback on the content and organization of the tests. For data analysis the one-way ANOVA test and post hoc multiple comparisons were used. The results demonstrated the feasibility and effectiveness of and students’ satisfaction with OSCE tests in the Zoom environment. However, more focus on communication techniques is required in a remote communication environment to better cover all patient health-related and drug communication aspects. There were identified differences between undergraduate students and practicing assistant pharmacists in assessing patients’ health problems and providing corresponding counseling. This result points to the need to implement the continuous development of patient-centered counseling techniques in the lifelong learning of pharmacists and the need to use innovative digital solutions, if applicable.

## 1. Introduction

Pharmacy education is changing to meet the healthcare needs of the public. The evolvement of pharmacy training has been driven by competency-based healthcare practices and the expanding clinical roles of pharmacy practitioners [1,2]. The role of the pharmacist is no longer limited to the preparation and dispensing of medicines, as the proportion of patient-centered care (e.g., patient education, preventative care, supporting patients with self-care) is growing [3,4]. Additionally, the continuous role of pharmacists in ensuring the effective and responsible use of medicines is essential and has advanced the pharmacy practice to be even wider [3,4,5]. The described aspects are important in pharmacists’ education to prepare a competent health workforce.

Changed expectations for pharmacy education also require a change in teaching and assessment methods. The multiple-choice test, traditionally used in Estonia, did not make it possible to assess students’ actual use of professional knowledge and communication skills. In health sciences, for clinical assessment, the Objective Structured Clinical Examinations (OSCEs) are one of the most widely used methods allowing students to use a variety of knowledge, such as pharmacotherapy, problem solving, and communication skills [6,7,8]. The OSCE test can be used to assess how the students are managing with different real-life situations and how they use clinical competences within given (limited) time—generally 3–5 min. The OSCE has been traditionally organized as a face-to-face test with several stations and cases. The OSCE content and scoring procedures are standardized, and in addition to the student, a patient and an assessor are involved in every station. Each OSCE participant experiences the same problem and is asked to perform the same task within the same timeframe [6,7,9]. The use of the OSCE is considered to be more objective, justified, and reliable than most other assessment methods [6].

The spread of COVID-19 and social-distancing rules challenged educators to find ways to replace classroom teaching and learning with web-based solutions. It gave a boost to develop new innovative e-learning platforms that use existing (web-based) tools or even create new ones [10,11,12]. In case of the OSCE, the challenges were even higher because of the multi-level approach and the involvement of different participants, i.e., simulated patients and assessors in every station.

The aim of this study was to assess whether and to what extent the transition from traditional face-to-face learning and assessment environment to the e-setting impacts the knowledge and skills acquired by students and their satisfaction with new solutions using the Objective Structured Clinical Examination (OSCE) test.

## 2. Materials and Methods

### 2.1. Study Context

The University of Tartu (UT) is the only university in Estonia providing an integrated bachelor and master program in pharmacy. The pharmacy curriculum was last updated in 2019 with a greater emphasis on the development of practical clinical and communication skills that are crucial in patients’ education and counseling about medicinal products. Additionally, new study and assessment methods, such as the OSCE and reflective practices, have been introduced [13].

The OSCE test is carried out at the fourth year (8th semester) during the “Social Pharmacy and Medicines Safety” course to assess the acquired competence about counseling of over-the-counter medicines and self-care aspects. The second time the OSCE testing is performed is in the fifth year (10th semester) after the traineeship period at a community pharmacy for the examination of clinical knowledge and communication skills for counseling of medicines and other pharmacy goods. All students receive individual case-by-case feedback. To pass the test, students have to collect at least 71% of the maximum points at all stations. If the score is below the required level, the students will have an additional interview with a social pharmacy lecturer to go over the shortcomings of the counseling. The study described in this article is limited to the results of the OSCE tests evaluating over-the-counter and self-medication counseling.

### 2.2. Organization of the OSCE Tests

Under normal circumstances, the multi-station (up to 6) OSCE tests were set up in the reorganized lecture room at the UT. All fourth year students (about 20–25 students) participated in the test with 3 min per station to solve the assignment. According to a set schedule, every 10 min a new student entered the room, and he/she was rotated through the stations. The assessors and simulated patients received information about the cases a couple of days beforehand and additional short briefing about how to perform and assess the assignments that took place prior the test. For assessors, the structured evaluation list was developed by the OSCE assessment team (lecturers at the UT and practicing pharmacists). Students received commented results individually within couple of days after the test in Moodle. For their feedback, students used an internationally standardized and structured evaluation list immediately after they completed the test [14].

The COVID-19 pandemic affected traditional, i.e., in-person, learning and, urgently, new ways to connect with students during a time of social distancing were required. The UT switched to remote teaching in just one day, due to already implemented and used digital learning aids [15]. However, to continue with the OSCE test in new circumstances was not an easy task, mostly because students must be physically rotated in between stations and outsourced simulated patients with digital skills were required. As a first solution, a combined test of written case solving (in Moodle) and counseling of a randomly selected written cases using OSCE principles (in MS Teams) was tested during the first wave of COVID-19 in spring 2020. The students took multiple roles, being a pharmacist in one case and a patient in another. Students revealed in their feedback that the OSCE structure was helpful in the oral test but rather confusing in written portion. In addition, they pointed out that being in several roles does not create objective conditions for assessing their knowledge and skills.

**Figure 1:**
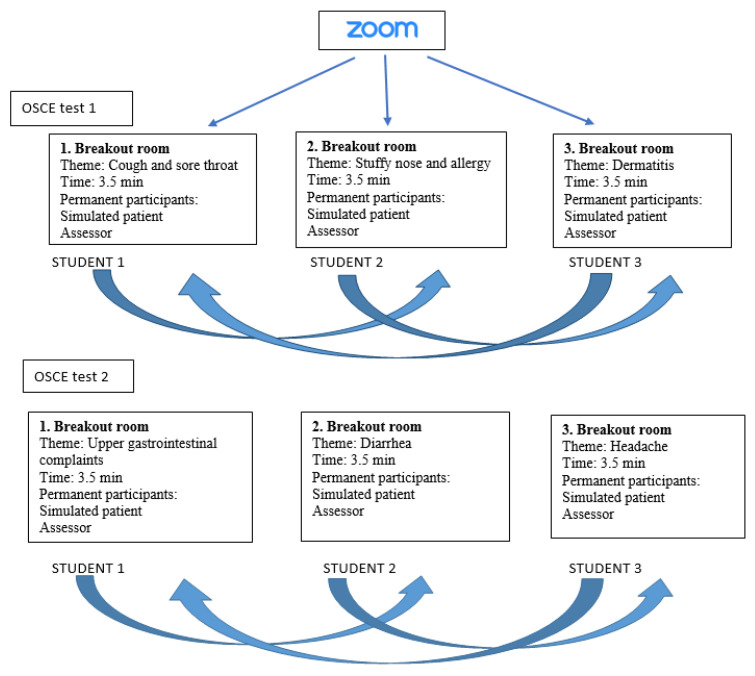
Detailed description of the performance of the OSCE test in Zoom.

In spring 2021, two online OSCE tests with three stations were executed in the Zoom platform enabling the use of ”break-out rooms“ as stations for private sessions with a selected number of people. In every room a different health topic was covered. Simulated patients and assessors were divided between rooms and stayed in one room until the end of the OSCE test, while the students were rotated between rooms/stations. In one session (15 min) three students were divided between rooms with one per station, and the student had 3.5 min to solve the case (Figure 1). The technical support was done by one of the assessors (allowing the students in Zoom and rotating between the break-out rooms). The same evaluation forms for assessors and feedback forms for students were used as in previous years. The assessors and simulated patients discussed the presentation and assessment of the cases online one week prior to the OSCE tests.

### 2.3. Study Sample and Design

The study compared the results of three face-to-face (2018–2019) and one electronically conducted (2021) OSCE tests, as well as students’ feedback on the performance of the tests. One randomly selected station was used to compare the results—the upper gastrointestinal complaints.

The traditional OSCE tests were conducted in spring 2018 and 2019 in the “Social Pharmacy and Drug Safety” course (4th year) and for assistant pharmacists within the continuous education program “From assistant pharmacist to pharmacist” in autumn 2019. The online OSCE test was performed in spring 2021 among fourth year pharmacy students during above mentioned course (Table 1).

During two OSCE tests, six different stations were applied. The students were in the role of pharmacists and had to advise the patient who turned to the pharmacy. The stimulated patients did not have any involvement with pharmacy and therefore required instructions before the OSCE tests. The assessors were lecturers of social pharmacy or practicing pharmacists (OSCE assessment team). Counseling was provided about different minor ailments and for different patient groups (age, gender, concomitant use of other medicines, etc.) (Figure 1).

### 2.4. Study Instrument

The evaluation list for assessors was developed by the OSCE assessment team. At each station, the student’s performance was assessed on seven topics: establishing and ending contact; evaluation of symptoms, concomitant symptoms, comorbidities, and medications used; treatment recommendations; drug information; appropriate language use; and general health and well-being counseling. Points were awarded on a four-step scale: no information was received—0; information partially provided—1; information mostly provided—2; all information provided—3. In addition, the simulated patient was able to provide three-step feedback (no—0; perhaps—1; yes—2) on whether he or she would like to contact this pharmacist in the future.

After passing all the stations, the students gave feedback on the test by answering a self-administered and internationally developed questionnaire [14]. Students were informed about the organization of the test and explained the need to collect feedback. By providing this information, students were considered informed about the study and gave their consent to participate in the study. Feedback was anonymous and voluntary.

### 2.5. Data Collection

Assessors’ evaluation sheets about upper gastrointestinal complaints, with no personal data and students’ anonymous feedback on the satisfaction and general organization of the test, were collected.

### 2.6. Data Analysis

For the analysis, the results of OSCE test—the case of upper gastrointestinal disorders and the students’ feedback, were separately entered into Microsoft Excel (Microsoft Corporation, Redmond, WA, USA) for initial data analysis. Data were imported to a Statistical Package for Social Sciences (SPSS^®^, IBM, Armonk, NY, USA), version 27. The average results of different study years were examined by the one-way ANOVA test and post hoc multiple comparisons, after checking the sample homogeneity of variances with the Levene’s test. The statistical significance level was set at *p* < 0.05. In the Results Section, the mean and SD are presented for statistically non-significant results, and the p-values of the post hoc comparisons for statistically significant results are reported. The full analysis (i.e., mean values for separate study years, F, and *p*-value) can be found in Table 2 and Table 3.

## 3. Results

### 3.1. Results of the OSCE Tests

The average total score obtained for solving the upper gastrointestinal problem varied from year to year but was not directly related to the environment in which the test was performed (Table 2). The mean results of the four studied groups were 2018, 16.17 ± 4.2 (70.3%); 2019, 18.79 ± 2.56 (81.7%); 2019 (assistant pharmacists), 16.95 ± 3.4 (74.3%); and 2021, 17.5 ± 3.63 (76.1%), respectively.

In initiating and ending the contact with the patient, the focus was on welcoming the patient into the pharmacy and establishing supportive and patient-centered environment. Additionally, inviting patients to return to the pharmacy in case of problems and politely ending the contact were considered. Compared to the previous years, in the online setting only half of the students were able to forward all the information (2019–2021 Tamhane, *p* = 0.004). However, when compared to the students’ groups, the stationary students provided all required communication in this section more than the practicing assistant pharmacists (2019–2019_AP Tamhane, *p* < 0.001).

In the identification of the symptoms, the highest score was reached in the online setting (2.43 ± 0.63) and the lowest for assistant pharmacists’ group (2.09 ± 0.67). By contrast, communication about concomitant illnesses and used medicines was the lowest in the online setting (1.75 ± 1.04): more than half of the students did not ask or asked information partially. The highest proactivity in identifying these aspects was demonstrated by pharmacy students in 2019 (2.47 ± 0.64).

In collaboration with the patient, an appropriate medicine or treatment should be selected during counseling. The assistant pharmacists demonstrated their expertise and practical knowledge (2019_AP–2021 Tukey, *p* < 0.001), and the students who used online setting were less confident (2019–2021 Tukey, *p* = 0.047).

Another important aspect in counseling the patient is providing correct and adequate information about the use and storage of medicines. Throughout the different years this part of counseling has improved (2018–2019_AP Tukey, *p* = 0.032; 2018–2021 Tukey, *p* = 0.047). A similar trend could be seen in language use to communicate with patients (2018–2019_AP Tukey, *p* = 0.009; 2018–2021 Tukey, *p* = 0.023).

An increasingly important issue is the patient’s general well-being and, therefore, aspects such as nutrition, regular workouts, and self-protection measures, e.g., hand hygiene, should be discussed. These improve patient health outcomes and general health knowledge. Assistant pharmacists paid the least attention to these aspects (2019_AP–2019 Tukey, *p* = 0.041). Surprisingly, students performed well in the online setting (2019_AP–2021 Tukey, *p* < 0.001), where almost 70% of students provided all the required information. Nevertheless, the patient feedback on the general provision of the counseling throughout years decreased and the OSCE tests that were performed in the online setting got the lowest results (1.57 ± 0.69); one-third of patients hesitated about going back to the particular pharmacist in the future. The result may indicate the need to balance the verbal and non-verbal aspects of teaching communication. Patients felt the absence or scarcity of the non-verbal communication and draw their own conclusions about the quality of counseling.

### 3.2. Students’ Feedback on the Provision of OSCE Tests

In general students were satisfied with the provision of OSCE test regardless of the environment in which it was carried out. Additionally, no statically significant differences were detected between student groups (Table 3), except students’ satisfaction with the available time to solve the cases in OSCE test (2018–2019; Tamhane *p* = 0.012).

## 4. Discussion

The COVID-19 pandemic has affected many areas of activities, including the provision of education. The entire education system has shifted from a traditional to an online education system [12]. Fast adaption with new rules and teaching environments was and still is a key to ensure qualified professionals in healthcare. To find novel ways to carry on daily teaching practices and modify existing methods is indispensable. Additionally, engaging students and asking for their feedback on the newly implemented teaching methods could highly affect the education outcomes [10].

Our study demonstrated the feasibility and effectiveness of conducting the OSCE tests in a Zoom environment; therefore, it could be considered as an attractive alternative to face-to-face OSCE tests. Alternatively, Thomas et al. assessed the patient counseling skills of pharmacy students using a virtual patient simulator. It showed some advantages regarding financial and logistical aspects in comparison with the traditional OSCE, but the simulation itself brought out some fundamental limitations, particularly on the quality and types of virtual patients [16]. Nevertheless, the technology-driven OSCE assessment provides the opportunity to continue to create cohesive, interactive dialogues between pharmacy students and simulated patients, thereby leveraging students’ confidence and their role in patient care in real-life situations. However, to create in an online setting of a supporting and accommodating patient-centered care environment is definitely a challenge. The results also revealed that to create welcoming and trustworthy contact with patient requires more input from students in an online setting. Although patient-centered communication skills and care aspects are taught in certain range within the pharmacy curricula, to implement them in an online setting is to some extent different. It is important to consider that while using online settings, it usually provides a clear picture and voice, but often lacks the intimacy and intricacy that real-world interactions possess [17]. Therefore, it is important to combine patient-centered attributes and digital (pharmaceutical) care aspects in pharmacy curricula to meet the needs of the society and to be able to provide patient-centered counseling through telecommunication tools [18,19,20]. Additionally, the International Pharmaceutical Federation reported the deficit in digital health training in pharmacy education. A skillful, competent, and digitally capable pharmaceutical workforce is required to make use of the full potential of digital health. Therefore, it is essential to integrate digital health into pharmacy education throughout the curriculum, but also to train the existing workforce with digital health skills. It can be assumed that knowing different digital environments and being digitally advanced relieves the stress related to the performance and assessment in a digital environment [12].

The importance of drug communication and the substantive quality of counseling in the provision of pharmacy services is growing over time [5,21]. This includes working with the patient to identify the patients’ medication needs and health concerns in order to provide them sufficient information for the effective and safe use of medicines or a suitable treatment plan. To start with effective consultation, the symptoms and concomitant illnesses, as well what medicines the patient has already used must be identified. In the online setting, the performed OSCE test revealed statistically significant gaps in the above-mentioned aspects. This could be explained with the irregular study period due to COVID-19, high-stress due to new examination method, or low adaptability to the new environment. However, information sharing about general well-being was statistically significantly higher in comparison with the assistant pharmacists’ performance. This could be elucidated with recent changes in the pharmacy curriculum at the UT. In comparison with previous curricula, the existing program includes more medicinal subjects and interactive study methods, whereas patient care and clinical competencies are more in focus [13,22]. The comparison of the test results of different student groups unexpectedly demonstrated poor performance in a number of the OSCE test sections of assistant pharmacists, although they have previous practical experience in providing community pharmacy services and having real-life contacts with the patients. They presented limited skills and competencies of making contact with patients and creating a trustworthy patient-centered environment, also determining the patient’s needs and educating about general health aspects. It highlights the need to acquire professional competencies not only during undergraduate studies, but also to follow life-long learning principles to advance professional development in daily practice. Despite the fact that in Estonia the continuous professional training of pharmacists has become more systematic in recent years, and some of the trainings can also be completed online, in the future, more teaching methods involving pharmacists (e.g., self-reflection and documentation as well as skills demonstration by simulated practice—the OSCE) should be applied [21]. This would require more effort from the learner but ensure high-quality professional development. Consequently, it is important to evolve practice-based competency standards to support continuous professional development activities and impact the quality of pharmacy practice [23,24].

Students’ feedback on the provision of the OSCE made it possible to assess the content and organization of the test. In general, students were satisfied with the OSCE test and ranked it as a “very good“ or “good“ learning method. The highest dissatisfaction was related with the limited timeframe (3–3.5 min per station) and high-stress factor. OSCE-related increased stress and anxiety could be caused by different and not before experienced examination processes and requirements, but also by the involvement of third parties, namely, assessors and simulated patients [25]. Different studies have shown that implementation of stress reduction technique prior to the OSCE would improve students’ performance by maximizing their learning and practical skills [26,27]. This is something to consider in the future in order to effectively assess the knowledge, attitude, and performance of the students.

In conclusion, our study showed the feasibility and effectiveness of and students satisfaction with the OSCE tests in a Zoom environment. More focus on communication techniques is required in a remote communication environment to better cover all patient health-related and drug communication aspects. Additionally, continuous professional development activities among practicing professionals are vital to ensure the quality of provided patient-centered care.

## Figures and Tables

**Table 1:** Distribution of the OSCE test participants and the applied settings 2018–2021.

Year	2018 (4th Year Students)	2019 (4th Year Students)	2019 (Assistant Pharmacists)	2021 (4th Year Students)
Number of participants (n)	12	15	23	28
Setting	Auditorium	Auditorium	Auditorium	Zoom

**Table 2:** Results of the OSCE among pharmacy students in different years and settings.

	2018 Mean (0–3; 0–2) ± Std	2019 Mean (0–3; 0–2) ± Std	2019_AP Mean (0–3; 0–2) ± Std	2021 Mean (0–3; 0–2) ± Std	Total Mean (0–3; 0–2) ± Std	One-Way ANOVA
Creating a contact/ending a contact	2.50	2.93	2.04	2.50	2.45	F(3, 74) = 7.65, *p* ≤ 0.001
	± 0.67	± 0.26	± 0.71	± 0.51	± 0.64	
Evaluating symptoms	2.17	2.40	2.09	2.43	2.28	F(3, 74) = 1.52, *p* = 0.217
	± 0.58	± 0.63	± 0.67	± 0.63	± 0.64	
Evaluating concomitant illnesses, medicines in use	2.17	2.47	2.26	1.75	2.10	F(3, 74) = 2.46, *p* = 0.069
	± 0.83	± 0.64	± 0.92	± 1.04	± 0.93	
Recommending medicines or treatment	2.25	2.33	2.65	1.82	2.23	F(3, 74) = 8.19, *p* ≤ 0.001
	± 0.62	± 0.62	± 0.49	± 0.67	± 0.68	
Giving drug information	1.50	2.00	2.39	2.25	2.13	F(3, 74) = 3.47, *p* = 0.020
	± 1.17	± 1.0	± 0.58	± 0.70	± 0.86	
Using appropriate language to the patient	2.25	2.67	2.83	2.75	2.68	F(3, 74)= 3.88, *p* = 0.012
	± 0.62	± 0.49	± 0.39	± 0.52	± 0.52	
Counseling health/ well-being issues	1.58	2.13	1.13	2.43	1.86	F(3, 74) = 7.37, *p* ≤ 0.001
	± 1.24	± 0.99	± 1.01	± 0.96	± 1.15	
Patient’s feedback	1.75	1.87	1.70	1.57	1.69	F(3, 74) = 0.87, *p* ≤ 0.461
	± 0.62	± 035	± 0.56	± 0.69	± 0.59	

**Table 3:** Students’ feedback on the provision of the OSCE test.

	2018 Mean (0–2; 0–4) ± Std	2019 Mean (0–2; 0–4) ± Std	2019_AP Mean (0–2; 0–4) ± Std	2021 (Zoom) Mean (0–2; 0–4) ± Std	Total Mean (0–2; 0–4) ± Std	One-Way ANOVA
The OSCE test enabled us to use clinical knowledge and communication skills	1.83 ± 0.38	1.91 ± 0.30	1.90 ± 0.30	1.80 ± 0.41	1.85 ± 0.36	F(3, 71) = 0.43, *p* = 0.731
The time to solve the OSCE test cases was sufficient	1.11 ± 0.74	1.82 ± 0.41	1.25 ± 0.91	1.58 ± 0.65	1.41 ± 0.76	F(3, 70) = 3.04, *p*= 0.034
The test was well conducted and ran smoothly	1.88 ± 0.33	2.00 ± 0.00	1.86 ± 0.36	1.96 ± 0.20	1.92 ± 0.28	F(3, 70) = 0.96, *p* = 0.418
Sufficient information was provided prior to the test	1.95 ± 0.23	2.00 ± 0.00	2.00 ± 0.00	2.00 ± 0.00	1.99 ± 0.12	F(3, 71) = 0.98, *p* = 0.406
All assessed knowledge was previously taught	1.84 ± 0.50	2.00 ± 0.00	1.95 ± 0.21	1.76 ± 0.44	1.87 ± 0.38	F(3, 73) = 1.60, *p* = 0.197
The OSCE made it possible to learn through real-life situations	1.89 ± 0.32	2.00 ± 0.00	1.71 ± 0.56	1.76 ± 0.60	1.82 ± 0.48	F(3, 72) = 1.13, *p* = 0.341
The OSCE caused less stress than other exams	1.20 ± 0.77	1.31 ± 0.86	0.68 ± 0.78	1.00 ± 0.83	1.01 ± 0.82	F(3, 75) = 2.18, *p* = 0.098
The OSCE test highlighted gaps in knowledge and communication skills	1.72 ± 0.46	2.00 ± 0.00	1.65 ± 0.59	1.75 ± 0.53	1.75 ± 0.49	F(3, 69) = 1.24, *p* = 0.301
Overall assessment of the OSCE test	3.19 ± 0.81	3.62 ± 0.51	3.22 ± 0.60	3.16 ± 0.69	3.26 ± 0.68	F(3, 78) = 1.49, *p* = 0.225

## Data Availability

Not applicable.
